# Qualitative colorimetric analysis of a Ir(iii)/Eu(iii) dyad in the presence of chemical warfare agents and simulants on a paper matrix†

**DOI:** 10.1039/c9ra00824a

**Published:** 2019-03-06

**Authors:** Genevieve H. Dennison, Christophe Curty, Alexander J. Metherell, Eva Micich, Andreas Zaugg, Michael D. Ward

**Affiliations:** Land Division, Defence Science and Technology Group Fishermans Bend Melbourne Australia Genevieve.Dennison@dst.defence.gov.au; Organic Chemistry Branch, Federal Office for Civil Protection (FOCP), Spiez Laboratory Spiez Switzerland; Department of Chemistry, University of Sheffield Sheffield S3 7HF UK; Department of Chemistry, University of Warwick Coventry CV4 7AL UK m.d.ward@warwick.ac.uk

## Abstract

The addition of G- and V-series organophosphorus chemical warfare agents and simulants to a paper-based assay of a dual-luminescent Ir(iii)/Eu(iii) dyad generated different emissive responses between the classes and compound types. The emission responses are complex and based not only on altering the balance between red Eu(iii)-based and blue Ir(iii)-based luminescent components, but also incorporate other factors such as analyte volatility, concentration and UV absorption. The extent of this emission colour change was analysed colorimetrically and related to the change in RGB output over time.

## Abstract 2

A dual emissive Ir(iii)/Eu(iii) dyad in a paper-based luminescence assay for liquid chemical warfare agents demonstrated clear visual responses.

## Graphical abstract

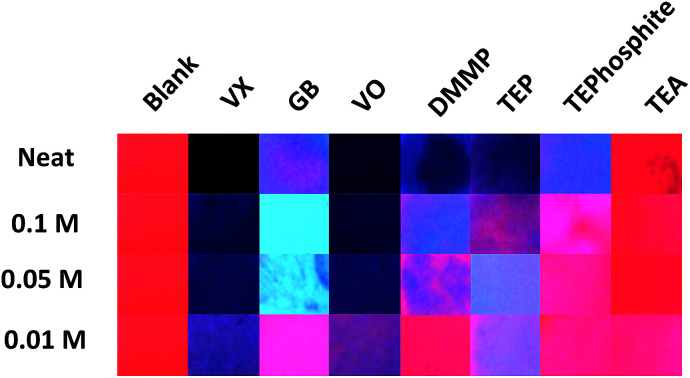

The traditional organophosphorus chemical warfare agents (OP CWAs) comprise two main series of agents: the G- and V-series (examples GB and VX respectively, Fig. 1). Although the G- and V-series OP CWAs display differing structural and physico-chemical properties (*e.g.* volatility), these chemicals are all fast-acting and potent acetylcholinesterase inhibitors that can cause incapacitation or death rapidly upon exposure.^1^ Thus, fast and reliable chemical sensing methods are required to inform and protect military and national security first responders and the general public.

**Fig. 1:**
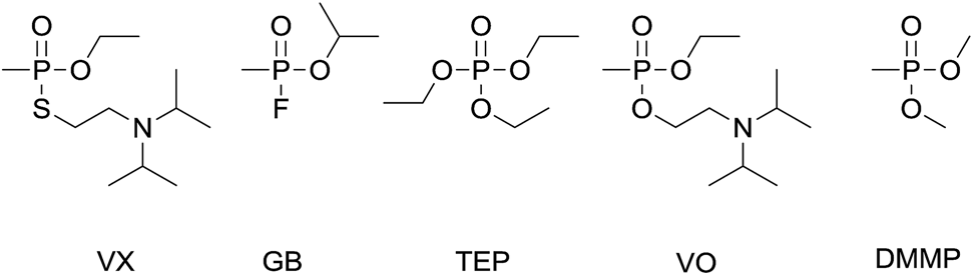
The chemical structures of the G and V-series chemical warfare agents VX (2-diisopropylaminoethyl ethyl methylphosphonothiolate) and GB (Sarin, isopropyl methylphosphonofluoridate) and simulants TEP (triethyl phosphate), VO (2-diisopropylaminoethyl ethyl methylphosphonate) and DMMP (dimethyl methylphosphonate) utilised in this investigation.

Trivalent lanthanide [Ln(iii)] complexes that display characteristic strong luminescence in the visible region are being increasingly exploited for the luminescence-based sensing of OP CWAs due to their known ability to form coordinative bonds with phosphonyl and phosphoryl moieties.^2^ In addition, the trivalent lanthanide complexes display high intensity and narrow emission bands, long excited state lifetimes and substantial Stokes shifts which provide attractive foundations for luminescence-based sensing systems.^3^

Previously we reported the solution-state emissive behaviour of an Ir(iii)/Eu(iii) dyad (denoted Ir·L·Eu, Fig. 2) as the basis of a ratiometric sensor for the V-series simulant 2-diisopropylaminoethyl ethyl methylphosphonate (VO).^4^ Upon excitation of this Ir·L·Eu dyad, photoinduced energy transfer from the ^3^MLCT/^3^LC excited-state of the Ir(iii) complex to the lower-lying ^5^D_0_ excited state of the Eu(iii) ion results in the sensitised red luminescence from the Eu(iii) centre with concomitant partial quenching of the Ir(iii) blue emission.^5^ Titration of VO aliquots into a solution of Ir·L·Eu resulted in a sequential colour change of the emission from red through to blue (ESI Fig. S1(a)†).^4^ This emission colour ‘switch’ is the result of both static and dynamic luminescence quenching of the Eu(iii) complex arising from the presence of VO. In particular, bidentate chelating coordination of VO to the Eu(iii) ion displaces the {Eu(hfac)_3_} unit from the Ir·L·Eu dyad (Fig. 2). This resulted in the loss of sensitised Eu(iii)-based emission and restoration of the blue Ir(iii)-based emission.^4,6^ Additional quenching of any residual Eu(iii)-based emission occurs by photoinduced electron transfer from the tertiary amine unit of either chelated VO, or by collision with excess VO in solution (Scheme 1).^4,6^

**Fig. 2:**
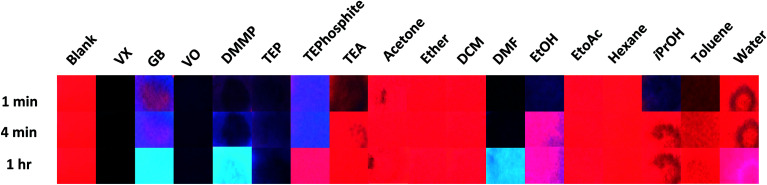
Comparison of the agent, simulant and potential interferent results over time using neat samples of analyte under 254 nm UV irradiation (one drop of each concentration added). Note the acetone 1 minute image is actually from the 30 second time point.

**Scheme 1:**
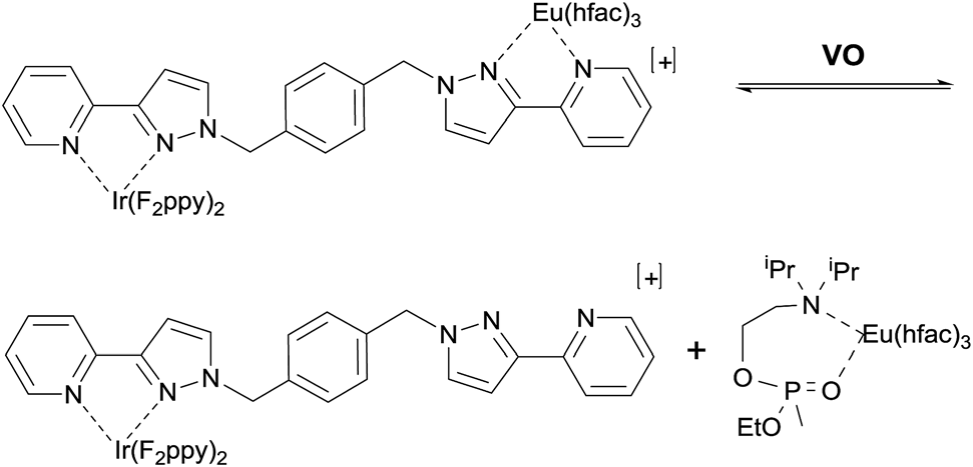
Interaction of the V-series simulant VO with the dyad sensor Ir·L·Eu.

There are numerous other literature examples of lanthanide-based sensors for the detection of OP CWAs, simulants, pesticides and toxic industrial chemicals.^2,6–14^ Whilst many of these systems have displayed positive results and properties in solution-based studies, only a few have been applied to solid state or matrix loaded assays such as polymers, films or sili*ca.* The translation of detection properties to solid state or matrix loaded systems is often imperative for application into electronic detection systems. However, there is also a need for simple presumptive systems such as colorimetric kits, disclosure sprays and detection papers which have the potential to be carried by numerous personnel (low burden and low cost) and utilised quickly and easily. These types of system generally work with high concentrations of analyte to give presumptive results indicative of exposure and contamination and to direct sampling activities.

Herein we investigate the use of qualitative luminescent paper-based assays of the Ir·L·Eu dyad for the detection and differentiation of different chemical warfare agents, simulants and potential interferents. **Warning**: the organophosphorus chemical warfare agents VX and GB are extremely potent acetylcholinesterase inhibitors that can cause incapacitation and death at extremely low concentrations. All of the work reported here has been performed by highly trained personnel in specialised facilities designed for the safe handling and experimentation with highly toxic chemicals. VX and GB are Schedule 1 chemicals under the Chemical Weapons Convention and their synthesis, use and experimentation is tightly controlled under national laws with international oversight form the Organisation for the Prohibition of Chemical Weapons in The Hauge.

The Ir·L·Eu dyad paper assays were prepared on Whatman 1 filter paper as described in the ESI.† One drop of a neat analyte was then placed on a test strip and images of the emissive colour response were taken with the test strip assays under 254 nm under UV irradiation at multiple time points (blank, immediate response, 30, 60, 120, 180, 240, 300 seconds and 60 minutes). This procedure was repeated for all CWAs, simulants and interferents studied. All CWAs and simulants were also tested at varied concentrations in MeCN (0.1 M, 0.05 M, 0.01 M and 0.001 M).

Two modes of visual comparison were performed to generate qualitative results: (i) a general comparison of all neat CWAs, simulants and interferents at varied time points to understand which systems would result in a visual change (Fig. 2); and (ii) a targeted comparison of the agent and simulant data at varied concentrations at a time point more suitable for in field testing (Fig. 3). The visual comparison of agent and simulant response over time using neat compounds (Fig. 2) generated some unexpected results in comparison to the solution studies with VO.^4^ VX and its simulant VO gave a very dark blue-black response that correlated well with each other over all time points. GB, however, displayed minimal colorimetric changes up to 5 minutes with the bright blue Ir(iii) luminescence seen only at the 1 hour time point (Fig. 2). This increased time of detection was initially attributed to a reactive pathway that can occur with the G-series CWAs only (hydrolysis of the P–F bond and subsequent detection of F^−^).^2,8^ However, when the similar (neat) results of the non-reactive G-series simulant DMMP (which does not contain a P–F bond) are considered this pathway is called into question. Whilst some competitive binding (Scheme 1) likely occurs with VX, VO, GB and DMMP at early time points, the excess analyte also absorbs the UV-light.^15^ Over time the excess of the more volatile analytes is lost *via* evaporation leaving the light blue Ir(iii)-based emission seen with GB and DMMP. With VX and VO evaporation does not occur and a dark blue colour persists; a similar result was also observed with the simulant TEP. This contrast (between the V- and G-series results) is likely the result of a combination of factors including differing volatilities of the CWAs (affecting UV Vis absorbance of excess analyte and test strip dampness), the presence of additional luminescence modulating moieties (*i.e.* amine in VX and VO) and the loss of luminescence *via* other pathways. The amine simulant TEA and the P(iii) simulant TEPhosphite did not generate a comparable result to the V- or G-series agents. Both generated near-reversible responses (TEA by 4 minutes and TEPhosphite at 1 hour) as a result of their volatilities and inability to form strong coordinative bonds to the Eu(iii) centre. The difference in emission intensity of the TEPhosphite assay compared to the other analytes at 1 minute and 4 minutes indicates that the system could be utilised to help distinguish between different classes of OP compounds.

**Fig. 3:**
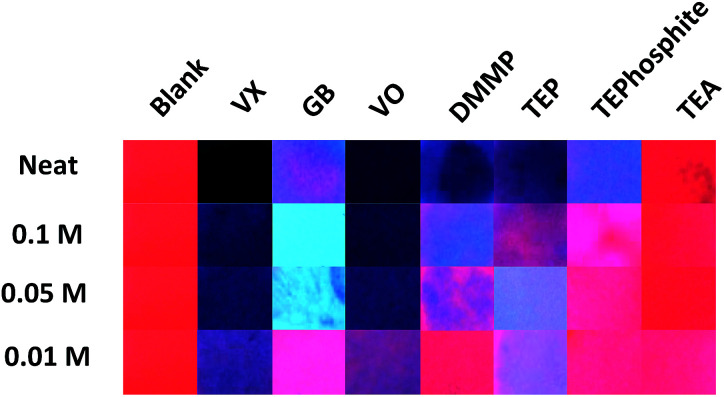
Comparison of the agent and simulant luminescence results at varied concentrations in MeCN (one drop of each concentration added) 4 minutes after exposure, under 254 nm UV irradiation.

The interferents tested were selected to cover a range of functional groups and volatilities as well as ubiquitous chemicals including common solvents and agent precursors (such as isopropanol). Numerous potential interferents either gave little to no response (*e.g.* water, hexane and DCM) or a rapid regenerative response as a result of the volatility of the analyte (ethanol, isopropanol, triethyl amine, diethyl ether and ethyl acetate). Toluene, acetonitrile and acetone, whilst resulting in a reversible darkening of the test assay upon application did not yield a change towards blue emission and were not considered to generate an ‘interferent like’ response. The major positive interferents identified were dimethyl formamide (DMF) and the alcohols. DMF displayed results that could be visually confused with both V- and G-series results (depending on the time point) with the initial dark colouration associated with the V-series results and the development over time of the bright blue luminescence similar to that noted with the G-series.

One of the main complications in using lanthanide complexes as sensors is the known interference of OH oscillators such as water and alcohols which efficiently provide non-radiative deactivation of the excited states of trivalent lanthanide complexes.^16^ The addition of ethanol and isopropanol generated a change from the initial red to purple/blue emission that almost completely visually reversed over time as a result of evaporation (Fig. 2). When water was tested a droplet formed on the surface of the test assay that left an emissive blue ring on absorption. Ultimately evaporation of the water yielded only a slight reduction in the visually observable red emission (Fig. 2). This lack of interference is especially favourable for presumptive tests to be used in operational environments where water is commonly encountered.

Presumptive tests taking 1 hour are well outside of operational requirements in the field. However, the time to detection could be lowered in this assay by decreasing the concentration of the analyte or *via* the application of heat or airflow to facilitate evaporation of excess analyte. The use of such processes to accelerate the development of the results is not unprecedented in analytical chemistry (*e.g.* TLC plate development) and with this in mind we focused on sample dilution and performed the visual comparison on the images taken 4 minutes after exposure (Fig. 3). This timeframe was selected to allow any residual solvent from the diluted samples to evaporate and to allow for the development of emission changes whilst still falling within the operational requirements for fast detection (where time for detection should be no longer than 4 minutes).^2,17^ The comparison of agent response *versus* concentration at 4 minutes (Fig. 3) demonstrates that the detection limit for visually interpretable responses is quite high (10–50 mM) which implies that the system is best suited for presumptive testing of high concentration or undiluted suspect liquid samples. Across all the concentrations tested, the differences in assay emission between the G and V-series agents was again noted. The VX and VO results were visually consistent (with each other) over neat, 0.1 M and 0.05 M tests even though the amount of analyte was reduced indicating that other factors rather than excess analyte contribute to the loss of luminescence. The bright blue Ir(iii) based emission generated with GB at 0.1 M and 0.05 M was not observed in the experiment using neat GB at 4 minutes. This blue emission is at least partially due the absence of the large excess of (neat) analyte. The results of the diluted G-series simulants DMMP and TEP are quite different to those obtained with GB and supports earlier observations that there may be a reactive component to the GB colour change mechanism (*i.e.* P–F hydrolysis and detection of F^−^). This means that at 0.1 M and 0.05 M GB can be readily differentiated from compounds that are sufficiently structurally similar to be used as simulants. TEA and TEPhosphite only generated luminescence colour changes when used neat, and not with dilute solutions, which is likely the result of poor binding affinity to Eu(iii) and (in the case of TEA) rapid evaporation.

Whist many of the analytes gave emission changes that can be observed with the naked eye (under UV light illumination); many of the responses are variations of the blue emission which may be too subtle to allow visual differentiation between OP compounds and other analytes. Thus interpretation of the results of these systems can be subjective and/or rely upon visual acuity in high stress situations. To combat this we analysed the photographic results to generate RGB colorimetric data for potential application into colour reading technologies to facilitate the reliable, fast and accurate interpretation of results in the field and to differentiate between the outputs obtained with various analytes. The images were processed as outlined in the ESI† and loaded into the colorimetric program Spot Finder 1.13 (iSense).^18^ An area in the centre of the spot formed upon liquid application with the most even colouration was selected for analysis and the averaged RGB data generated. The percentage change in intensity over time (300 seconds) for each RGB colour element at each concentration point was plotted to give a visual representation of the colorimetric changes occurring over the experiment. Fig. 4 demonstrates the RGB plots of the luminescence responses for the application of neat VX, VO, GB and DMMP over 5 minutes (300 seconds). From this analysis it can be seen that VX and VO generate greater colorimetric differences in the blue component compared to GB and DMMP. VX results in near complete loss of the red and green components of the dyad and majority (∼85% loss) of the blue component with no recovery of any colour component over the experiment time; whilst VO only demonstrates complete loss of the red component with significant recovery of the blue component after approximately 60 seconds. It should be noted that increases in the blue component are observable in VX and VO down to 0.01 M (ESI Fig. S4 and S8†). When looking at the G-series results, GB displays partial loss of the red component with a substantial increase in the blue component which is a trend observable down to 0.05 M (Fig. 4). DMMP however, displayed less significant increases in the blue component observed with GB at neat concentrations but demonstrated more when diluted (ESI Fig. S10†). This is again most likely due to the reduced amounts of excess analyte present at the lower concentrations.

**Fig. 4:**
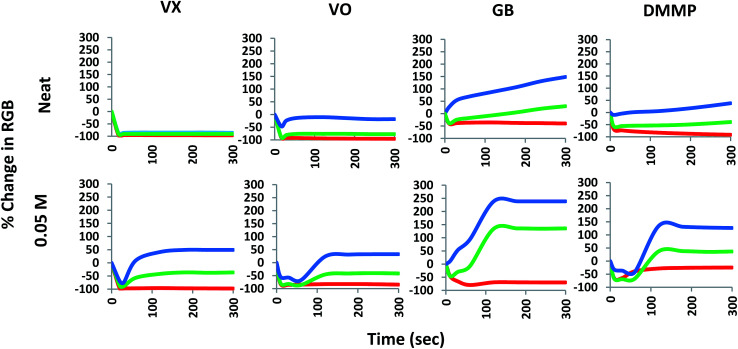
Colorimetric time response profiles (smoothed data) over 300 seconds by percentage change in each of the R, G and B colour components for neat and 0.05 M VX, VO, GB and DMMP.

## Conclusions

The luminescent dyad Ir·L·Eu paper assay, which contains both red and blue luminescent components, displays different luminescent responses in the presence of a variety of OP CWAs, simulants and interferents. The colorimetric (emission) responses are complex and based not only on altering the balance between red and blue luminescent components by a range of quenching mechanisms, but also incorporate other factors such as analyte volatility (wetting), concentration and UV absorption.

We have demonstrated that the Ir(iii)/Eu(iii) dyad paper assay for the presumptive detection of chemical warfare agents can be utilised, under UV-light excitation, to provide a clear visual response to differentiate between different CWA classes (G and V) as well as demonstrating some differentiation between the core functional groups of various phosphorus systems (phosphate, phosphite, phosphonate). The fast recovery of the red luminescence when exposed to many common interferents is promising as is the paper assay's resistance to significant interference from water.

Although signal development did take significant time for some neat analytes, we demonstrated that appearance of the GB signal could be hastened by dilution. It is also feasible that heat or airflow could be introduced to facilitate signal development in the future. RGB colorimetric analysis can be applied to differentiate between similar compounds (*i.e.* VX and VO) suggesting that the use of handheld colorimetric readers (with inbuilt UV light sources) may be a feasible way to overcome issues of visual acuity by providing more colorimetric information to reduce the likelihood of false positives from similar compounds. This luminescent dyad system could also be applied to fluorescent/colorimetric arrays that distinguish between many types of OP CWAs, simulants and interferents.

## Conflicts of interest

There are no conflicts to declare.

## Supplementary Material

RA-009-C9RA00824A-s001
