# Supplementary material for: Qualitative colorimetric analysis of a Ir(iii)/Eu(iii) dyad in the presence of chemical warfare agents and simulants on a paper matrix
Source: RSC Adv. 2019 Mar 6;9(14):7615–9. doi: 10.1039/c9ra00824a (PMC9061201; doi:10.1039/c9ra00824a)
Supplement: RA-009-C9RA00824A-s001 [file RA-009-C9RA00824A-s001.pdf]

Electronic Supplementary Material (ESI) for RSC Advances.
This journal is © The Royal Society of Chemistry 2019

Colorimetric luminescence-based analysis of chemical warfare agents and simulants using a dual emissive Ir(III)/Eu(III) dyad on a paper matrix.

Genevieve H. Dennison,[a]* Christophe Curty,[b] Alexander Metherell,[c] Eva Micich,[a] Andreas Zaugg[b] and Michael D. Ward[d]*

[a] Land Division, Defence Science and Technology Group, Fishermans Bend, Melbourne, AUSTRALIA.
[b] Federal Office for Civil Protection FOCP, Spiez Laboratory, Organic Chemistry Branch, Spiez, SWITZERLAND.
[c] Department of Chemistry, University of Sheffield, Sheffield , UNITED KINGDOM.
[d] Department of Chemistry, University of Warwick, Coventry, UNITED KINGDOM.

Materials and Methods:

Unless otherwise indicated, all reagents and solvents were obtained from commercial suppliers (Sigma–Aldrich, Alfa Aesar, Merck, VWR) and were used without further purification. The **Ir.L.Eu** dyad and the simulant VO were prepared as previously described.[1,2] The chemical warfare agents 2-[(diisopropylamino)ethyl]-O'ethyl methylphosphonothioate (VX) and O-isopropyl methylphosphonofluoridate (GB) were synthesised in-house, with characterisation data reported previously.[3]

Results and Data Analysis:

All photographs were obtained in a UV CM 10 Fluorogenic analysis cabinet (dark box) using a Canon EOS 500D camera with no flash. F-stop: f/5.6, Exposure time: 1/50 sec., ISO Speed: ISO-1600. The paper test strips were irradiated with a Spectroline ultraviolet light at 254 and 365 nm.

The dyad paper assays were prepared fresh daily by dipping strips of Whatman 1 filter paper into $1x10^{-3}$ M solutions of the Ir.L.Eu dyad (MeCN) in a petri dish until completely submerged. Using lower dyad concentrations resulted in little ($1x10^{-4}$ M) to no lanthanide-based ($1x10^{-5}$ M) luminescence under the Uv-Vis irradiation utilised (Figure S1b). Once removed from the solution the wet paper assays were dried for 1 hour at 30 – 35 ^{o}C and test strips with uneven distribution of dyad were discarded. One drop of a neat analyte was placed on a test strip and photographs of the emissive colour response were taken under UV irradiation at multiple time points (blank, immediate response, 30 seconds, 1, 2, 3, 4, 5 minutes and 1 hour). The photographs were taken with the test strips under both 256 and 365 nm. However, this testing methodology was repeated for all chemical warfare agents, simulants and interferents studies. All CWAs and simulants were also tested at varied concentrations (0.1 M, 0.05 M, 0.01 M and 0.001 M in MeCN).

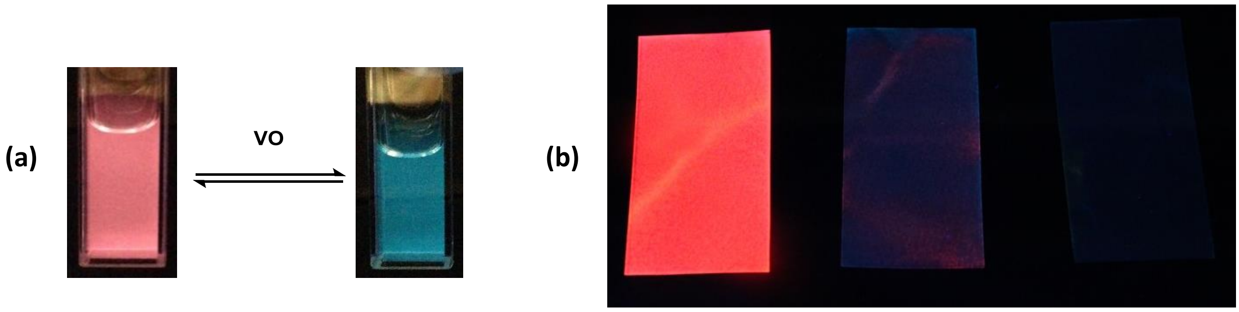

Figure S1: (a) The solution state colour change of the **Ir•L•Eu** dyad**,** from the red metal centred Eu(III) emission to the blue Ir(III) emission in the presence of VO, (b) photographs of dyad loaded Whatman 1 filter paper irradiated under 256 nm UV light. Left: 1 $x10^{-3}$ M in MeCN, Middle: 1 $x10^{-4}$ M in MeCN and Right: 1 $x10^{-5}$ M in MeCN.

A visual comparison of the resulting images taken under 254 and 365 nm UV irradiation demonstrated that a more even colouration was obtained using 254 nm irradiation. These samples also displayed far less discolouration due to residual paper dampness or complex movement on the paper assay than those visualised using 365 nm irradiation. Thus the use of 365 nm UV light for visualisation of the luminescence response was not investigated further.

All images for an individual experiment set (a single concentration of a particular analyte over every time point) were loaded into the software program Fiji.[4] The images were stacked and normalised so that each photo was lined up precisely with the others in the series (including a blank) and the images cropped to focus on the spot (see ESI). The cropped Fiji files were then loaded into the colorimetric program Spot Finder 1.13 (isense).[5] An area in the centre of the first spot (photograph of the immediate response) with the most even colouration was selected for analysis and the averaged RGB data generated. The percentage change in intensity over time (300 seconds) for each RGB colour element at each concentration point was plotted to give a visual representation of the colorimetric changes occurring over the experiment.

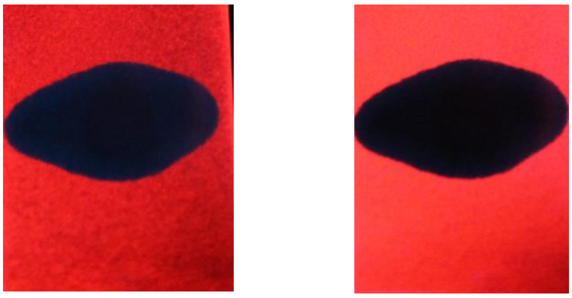

Figure S2: Photographs of the neat VX at 2 minutes sample under differing UV irradiation wavelength. Left: 365 nm and Right: 256 nm.

VX, 254 nm

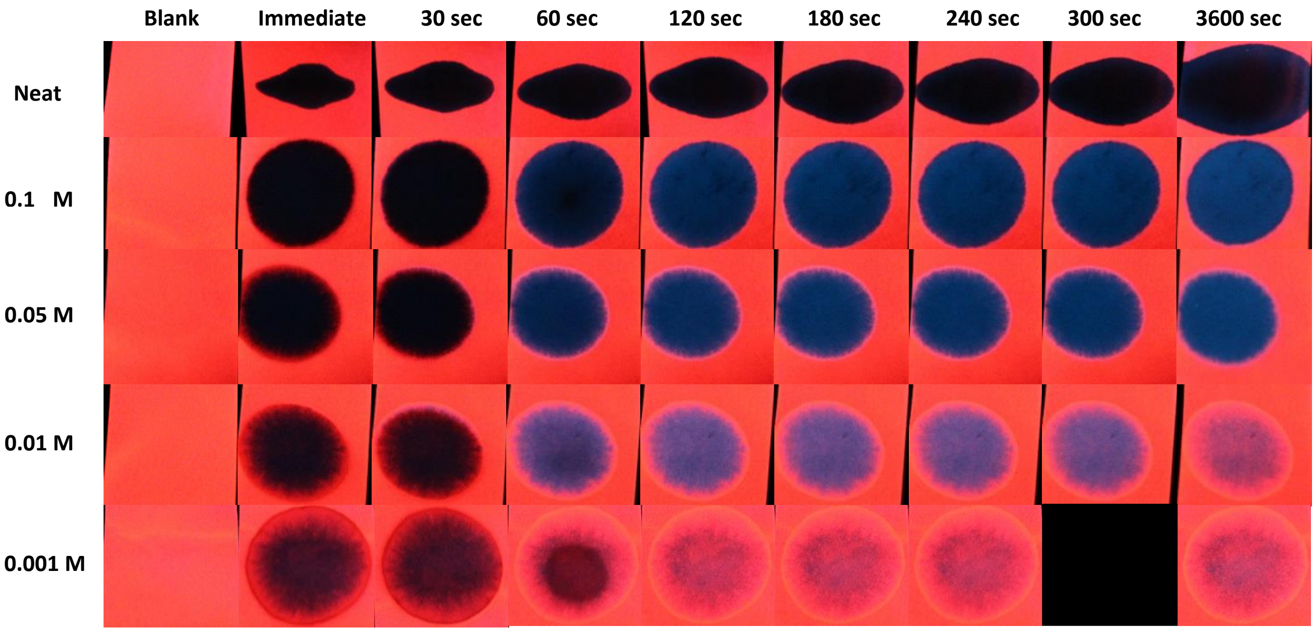

Figure S3: Normalised photographs of the dyad loaded paper assay with one drop of VX at varied concentrations over time. The image of 5 minute (300 sec) sample at 0.001 M failed or was corrupted and has not been displayed.

Table S1: RGB triplet data generated from the Spotfinder software for the normalised photographs from the VX experiments in Figure S3.

**Neat**	**Time**	**0**	**17**	**30**	**60**	**120**	**180**	**240**	**300**	**3600**
	**Red**	4080	192	192	192	176	176	160	160	288
	**Green**	1408	128	128	128	128	128	128	128	160
	**Blue**	1392	176	192	208	208	208	208	192	288
**0.1 M**		**0**	**23**	**30**	**60**	**120**	**180**	**240**	**300**	**3600**
	**Red**	4080	128	128	96	64	48	48	48	192
	**Green**	1328	144	144	288	576	656	656	672	880
	**Blue**	1152	288	256	592	1200	1328	1344	1360	1712
**0.05 M**		**0**	**20**	**30**	**60**	**120**	**180**	**240**	**300**	**3600**
	**Red**	4080	112	112	128	160	128	112	96	160
	**Green**	1184	144	128	512	688	752	736	752	912
	**Blue**	1008	304	256	1104	1424	1504	1504	1504	1744
**0.01 M**		**0**	**20**	**30**	**60**	**120**	**180**	**240**	**300**	**3600**
	**Red**	4080	432	528	1120	1552	1616	1680	1744	2816
	**Green**	1280	192	192	816	1136	1152	1168	1168	1008
	**Blue**	1056	384	368	1616	2112	2112	2112	2096	1680
**0.001 M**		**0**	**18**	**30**	**60**	**120**	**180**	**240**	**300**	**3600**
	**Red**	4080	1072	1168	1728	3296	3312	3312	[redacted]	3360
	**Green**	1344	304	288	288	800	832	832	[redacted]	960
	**Blue**	1392	608	576	544	1328	1360	1376	[redacted]	1536

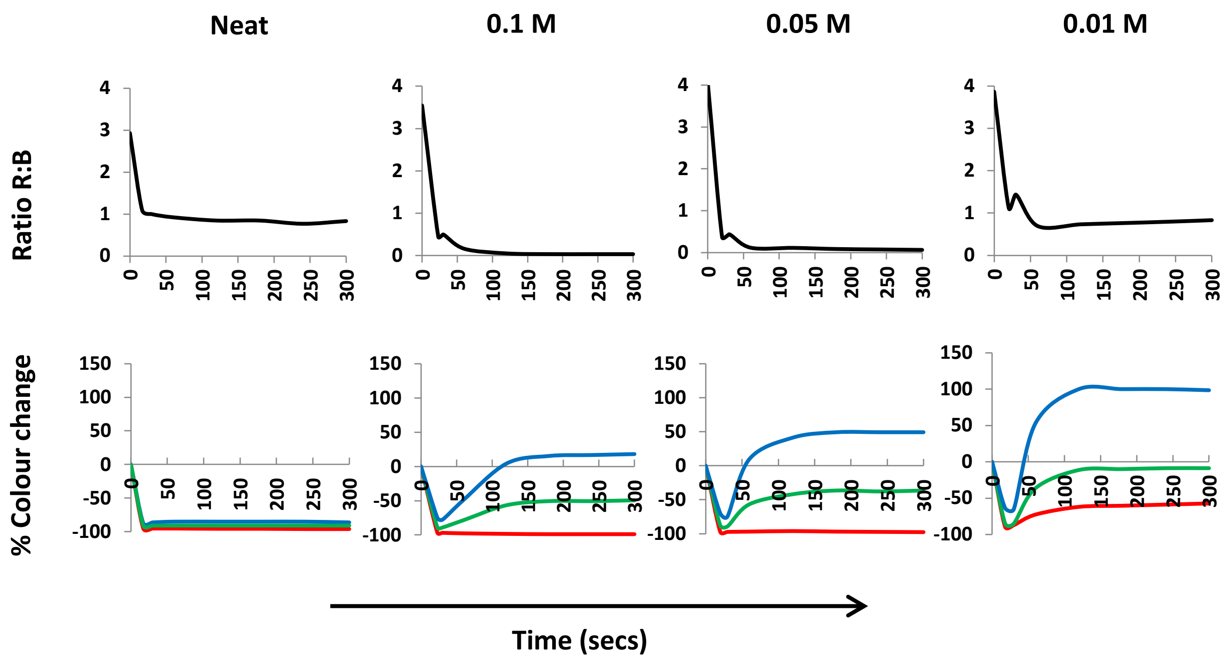

Figure S4: Colorimetric time response profiles over 5 minutes (300 seconds) by percentage change in each of the R, G and B colour components for VX at varied concentrations.

Sarin GB, 254 nm

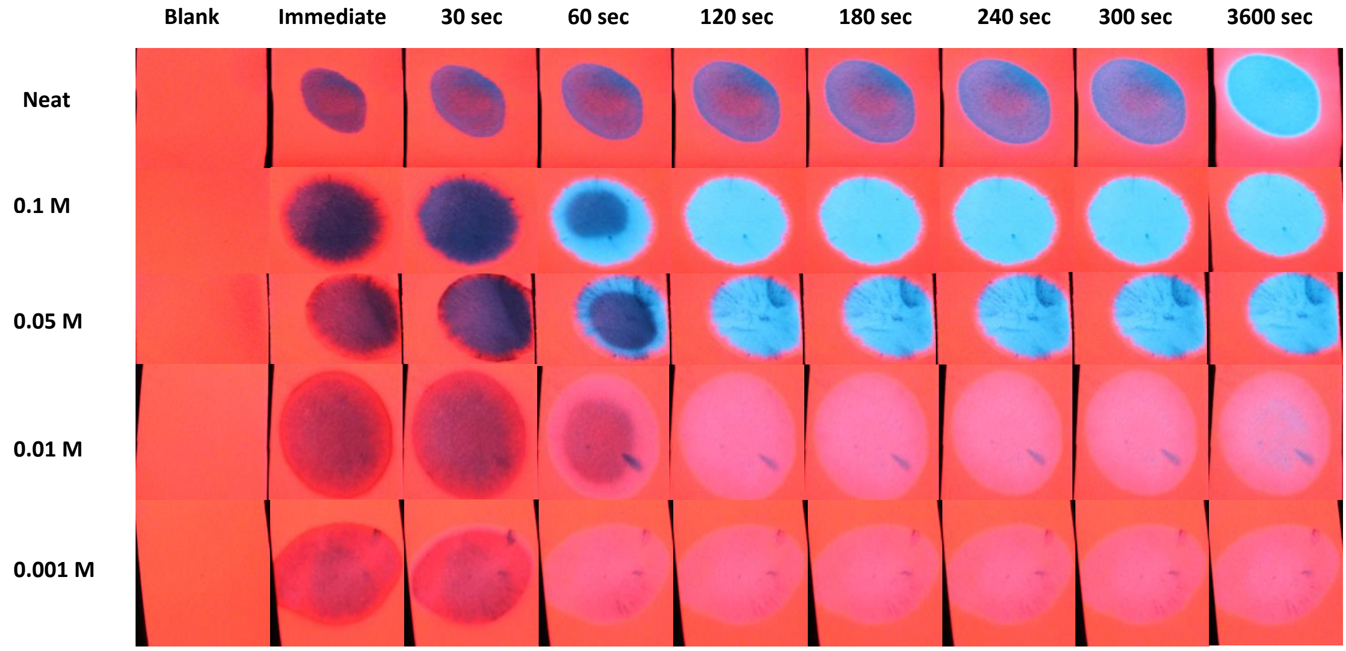

Figure S5: Normalised photographs of the dyad loaded paper assay with one drop of GB at varied concentrations over time.

Table S2: RGB triplet data generated from the Spotfinder software for the normalised photographs from the GB experiments in Figure S5.

		**0**	**11**	**30**	**60**	**120**	**180**	**240**	**300**	**3600**
**Neat**	**Red**	4080	2416	2512	2592	2624	2544	2512	2448	1312
	**Green**	1440	880	1088	1184	1344	1520	1728	1872	3392
	**Blue**	1232	1568	1904	2080	2320	2560	2848	3056	4080
**0.1 M**		**0**	**11**	**30**	**60**	**120**	**180**	**240**	**300**	**3600**
	**Red**	4080	1072	800	496	1648	1616	1632	1584	1664
	**Green**	1424	640	848	1680	3440	3456	3456	3456	3472
	**Blue**	1184	1280	1712	2848	4080	4080	4080	4080	4080
**0.05 M**		**0**	**11**	**30**	**60**	**120**	**180**	**240**	**300**	**3600**
	**Red**	4080	2160	1392	832	1248	1248	1232	1232	1248
	**Green**	1344	720	944	1232	3152	3168	3152	3168	3232
	**Blue**	1200	1344	1840	2320	4064	4064	4064	4064	4064
**0.01 M**		**0**	**20**	**30**	**60**	**120**	**180**	**240**	**300**	**3600**
	**Red**	4080	3040	2880	3472	4080	4080	4080	4080	3952
	**Green**	1536	752	848	1040	1904	1984	2032	2064	2176
	**Blue**	1408	1264	1472	1664	2751	2800	2800	2832	3024
**0.001 M**		**0**	**18**	**30**	**60**	**120**	**180**	**240**	**300**	**3600**
	**Red**	4080	3536	3680	4080	4080	4080	4080	4080	4080
	**Green**	1520	896	992	1632	1680	1712	1712	1744	1792
	**Blue**	1152	1408	1568	2240	2320	2352	2368	2432	2496

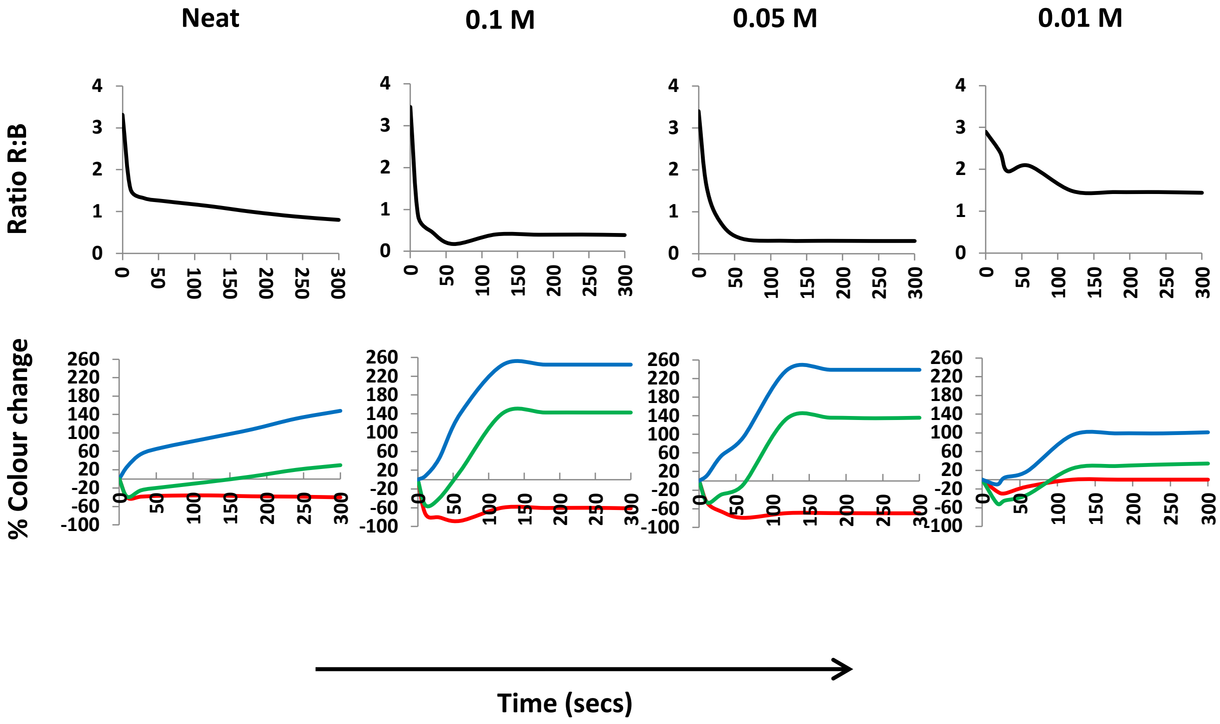

Figure S6: Colorimetric time response profiles over 5 minutes (300 seconds) by percentage change in each of the R, G and B colour components for GB at varied concentrations.

VO, 254 nm

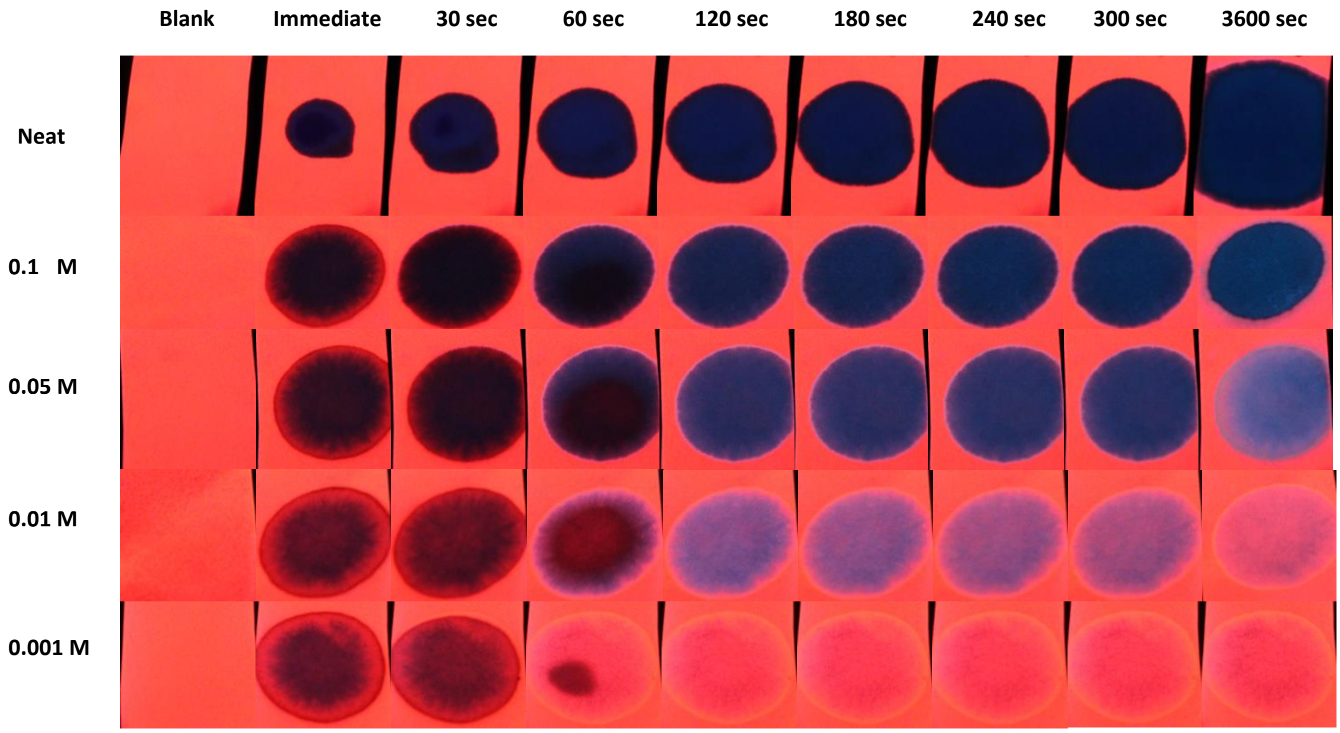

Figure S7: Normalised photographs of the dyad loaded paper assay with one drop of VO at varied concentrations over time.

Table S3: RGB triplet data generated from the Spotfinder software for the normalised photographs from the VO experiments in Figure S7.

		0	17	30	60	120	180	240	300	3600
**Neat**	**Red**	4080	352	336	304	240	208	192	176	80
	**Green**	1472	144	288	352	352	352	336	336	384
	**Blue**	1232	656	944	1072	1104	1056	1008	1008	1072
**0.1 M**		**0**	**10**	**30**	**60**	**120**	**180**	**240**	**300**	**3600**
	**Red**	4080	464	320	32	368	336	240	208	160
	**Green**	1344	224	192	208	528	576	608	608	752
	**Blue**	1168	464	416	432	1152	1216	1280	1280	1472
**0.05 M**		**0**	**10**	**30**	**60**	**120**	**180**	**240**	**300**	**3600**
	**Red**	4080	720	592	624	704	720	688	624	1280
	**Green**	1264	256	224	176	688	736	752	736	1296
	**Blue**	1136	512	480	368	1408	1488	1504	1504	2256
**0.01 M**		**0**	**8**	**30**	**60**	**120**	**180**	**240**	**300**	**3600**
	**Red**	4080	1456	1504	1824	2128	2304	2464	2592	3648
	**Green**	1168	272	256	208	1216	1232	1200	1184	1200
	**Blue**	992	544	512	400	2128	2096	2016	1936	1744
**0.001 M**		**0**	**9**	**30**	**60**	**120**	**180**	**240**	**300**	**3600**
	**Red**	4080	1552	1920	4048	4080	4080	4080	4080	4064
	**Green**	1344	304	320	864	944	960	944	944	928
	**Blue**	1120	624	592	1216	1264	1296	1280	1280	1264

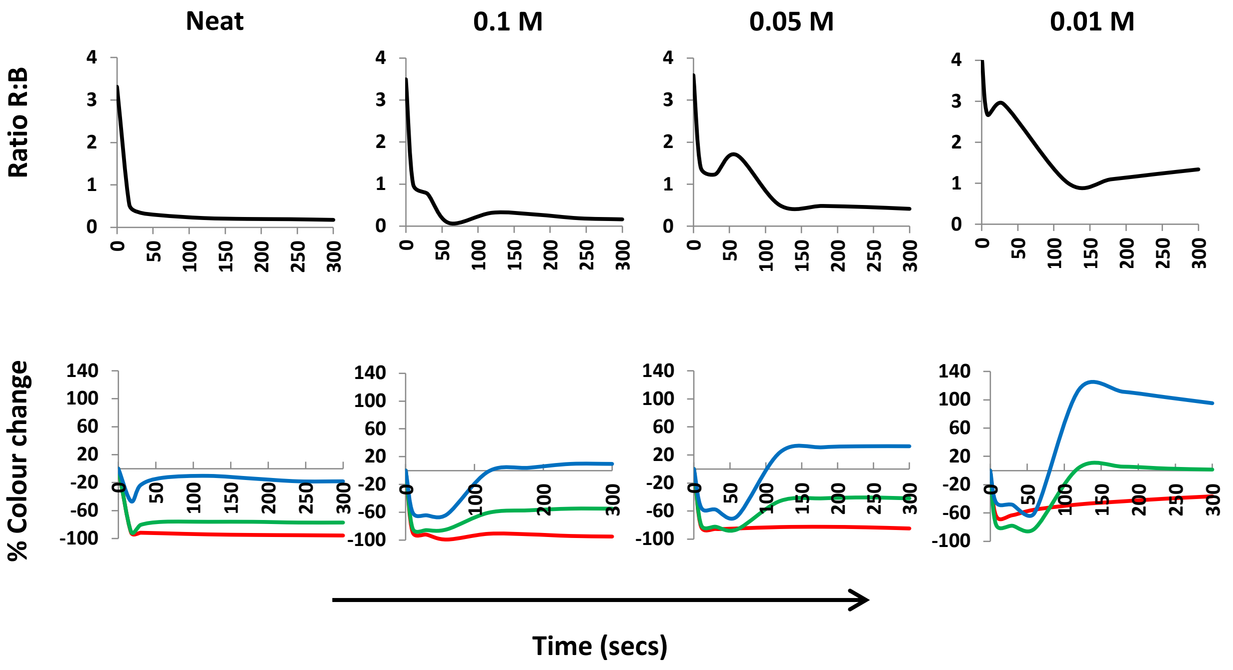

Figure S8: Colorimetric time response profiles over 5 minutes (300 seconds) by percentage change in each of the R, G and B colour components for VO at varied concentrations. Outlier removed from 0.01 M R:B ratio profile at 60 secs.

DMMP, 254 nm

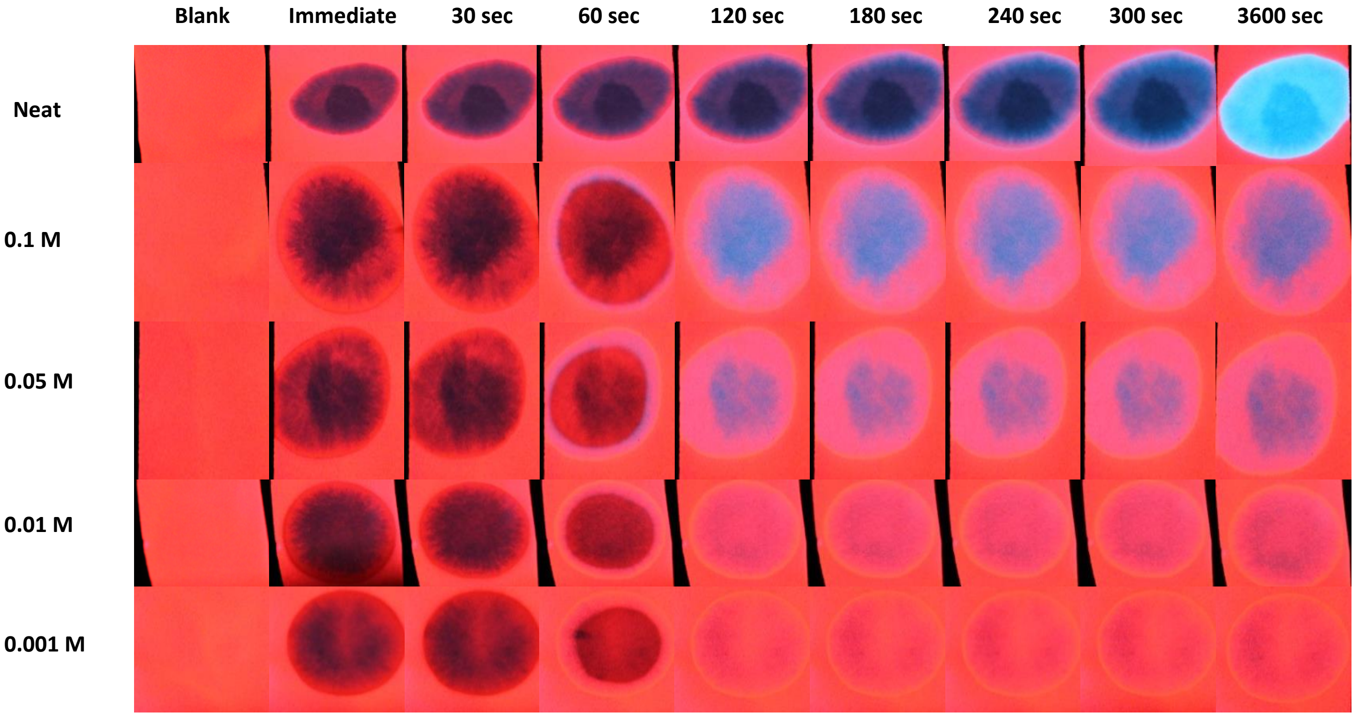

Figure S9: Normalised photographs of the dyad loaded paper assay with one drop of DMMP at varied concentrations over time.

Table S4: RGB triplet data generated from the Spotfinder software for the normalised photographs from the DMMP experiments in Figure S9.

**Neat**		**0**		**30**	**60**	**120**	**180**	**240**	**300**	**3600**
	**Red**	4080	1264	1072	896	688	544	432	336	640
	**Green**	1264	544	560	576	592	624	688	768	3184
	**Blue**	1136	1024	1088	1152	1200	1296	1424	1568	4080
**0.1 M**		**0**	**9**	**30**	**60**	**120**	**180**	**240**	**300**	**3600**
	**Red**	4080	1136	1136	1712	1856	1984	2032	2064	2064
	**Green**	1264	352	352	336	2032	1984	1984	1968	1648
	**Blue**	1120	720	704	624	3232	3184	3184	3152	2784
**0.05 M**		**0**	**12**	**30**	**60**	**120**	**180**	**240**	**300**	**3600**
	**Red**	4080	1360	1456	2480	2928	3024	3056	3072	2880
	**Green**	1136	368	352	368	1568	1568	1536	1552	1344
	**Blue**	1088	736	688	624	2560	2512	2480	2464	2208
**0.01 M**		**0**	**11**	**30**	**60**	**120**	**180**	**240**	**300**	**3600**
	**Red**	4080	1552	1680	2544	3984	3968	3968	3936	3904
	**Green**	1280	384	368	368	1088	1088	1104	1072	1024
	**Blue**	1200	688	704	640	1664	1680	1680	1648	1584
**0.001 M**		**0**	**9**	**30**	**60**	**120**	**180**	**240**	**300**	**3600**
	**Red**	4080	2192	2448	3152	4080	4080	4080	4080	4080
	**Green**	1376	416	384	384	1168	1152	1136	1120	1088
	**Blue**	1200	752	672	560	1344	1328	1328	1296	1280

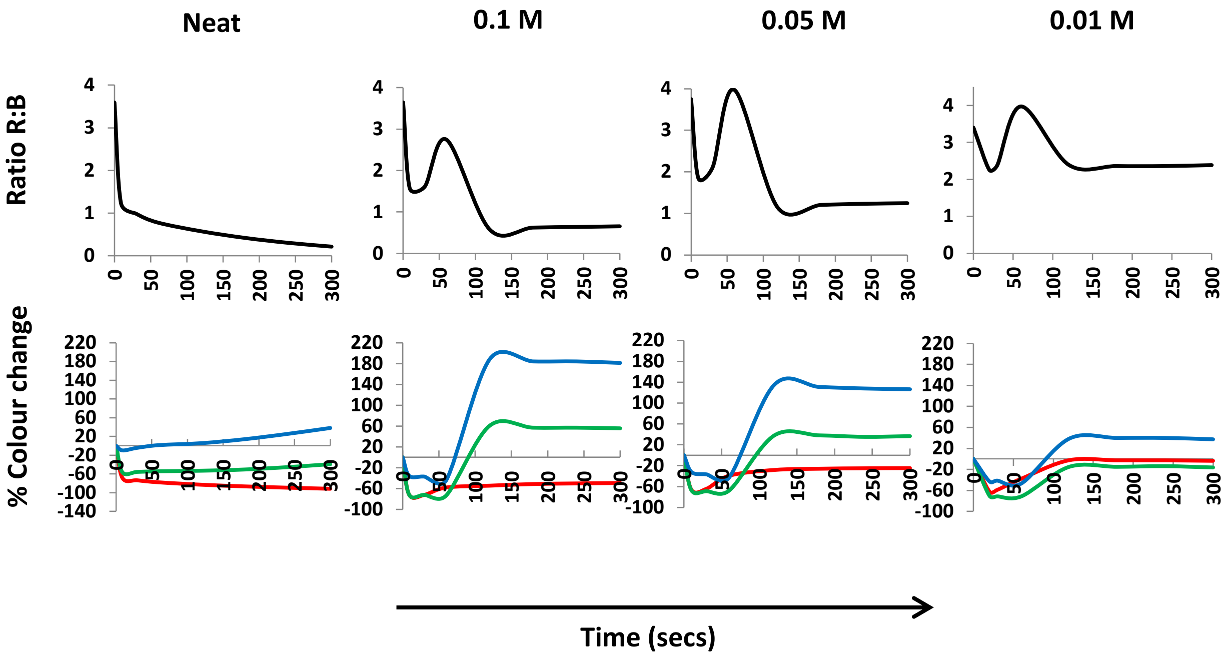

Figure S10: Colorimetric time response profiles over 5 minutes (300 seconds) by percentage change in each of the R, G and B colour components for DMMP at varied concentrations.

TEP, 254 nm

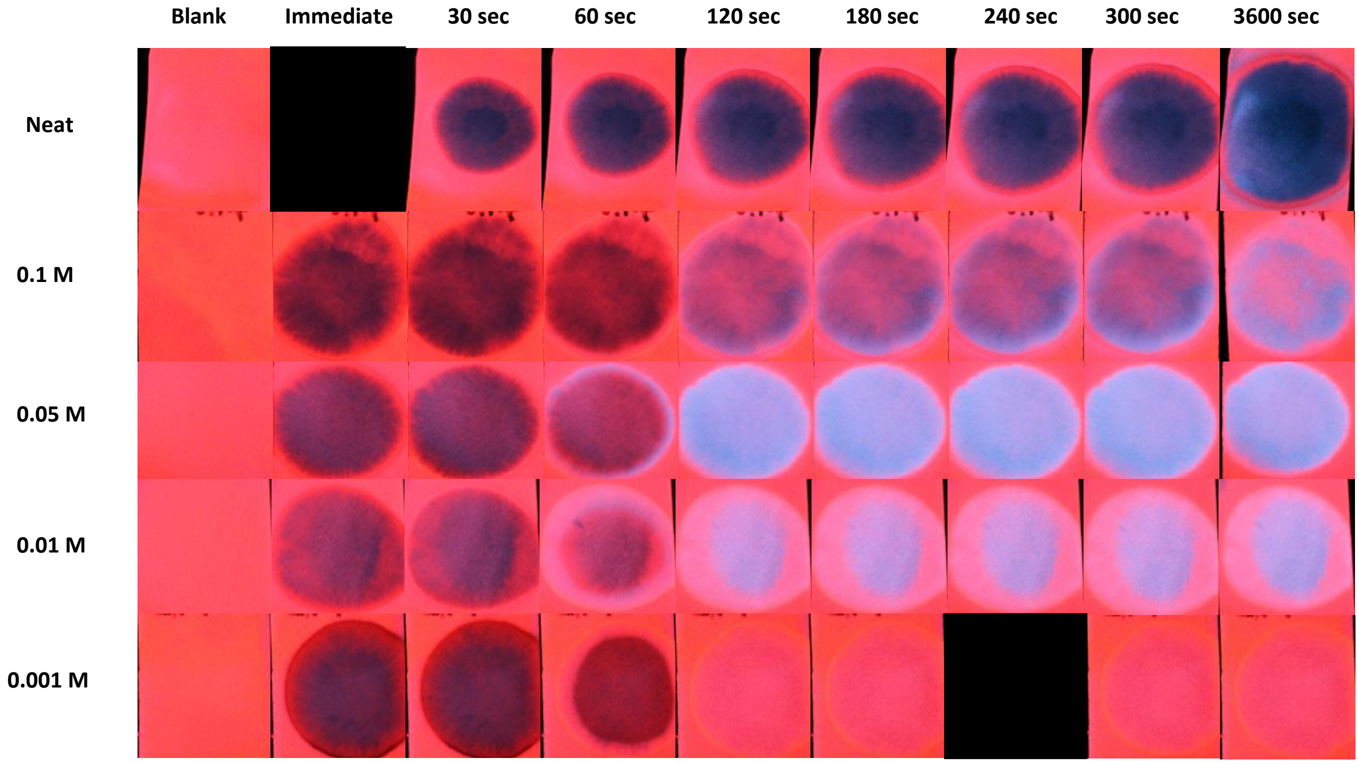

Figure S11: Normalised photographs of the dyad loaded paper assay with one drop of TEP at varied concentrations over time. The images immediately after addition of the neat TEP and 4 minutes (240 sec) at 0.001 M were corrupted and have not been displayed.

Table S5: RGB triplet data generated from the Spotfinder software for the normalised photographs from the TEP experiments in Figure S11.

**Neat**		**0**		**30**	**60**	**120**	**180**	**240**	**300**	**3600**
	**Red**	4080		1072	928	880	832	800	768	256
	**Green**	1328		640	640	704	704	720	736	656
	**Blue**	1696		1296	1312	1424	1440	1504	1520	1392
**0.1 M**		**0**	**9**	**30**	**60**	**120**	**180**	**240**	**300**	**3600**
	**Red**	4080	1856	1904	2400	3120	3040	2864	2800	3424
	**Green**	1248	432	416	416	1040	1184	1232	1328	1888
	**Blue**	1152	832	784	768	1808	2064	2176	2320	2912
**0.05 M**		**0**	**12**	**30**	**60**	**120**	**180**	**240**	**300**	**3600**
	**Red**	4080	2160	2224	2784	2752	2800	2816	2816	2880
	**Green**	1296	704	752	800	2496	2608	2624	2624	2560
	**Blue**	1584	1344	1424	1488	3760	3808	3824	3824	3760
**0.01 M**		**0**	**11**	**30**	**60**	**120**	**180**	**240**	**300**	**3600**
	**Red**	4080	2352	2208	2800	3312	3264	3216	3248	3040
	**Green**	1360	784	880	896	2336	2368	2400	2448	2544
	**Blue**	1616	1472	1632	1632	3520	3552	3568	3632	3712
**0.001 M**		**0**	**9**	**30**	**60**	**120**	**180**		**300**	**3600**
	**Red**	4080	1632	1744	2464	4064	4064		4064	4064
	**Green**	1328	576	560	448	1168	1168		1184	1184
	**Blue**	1376	1120	1072	832	1760	1760		1776	1776

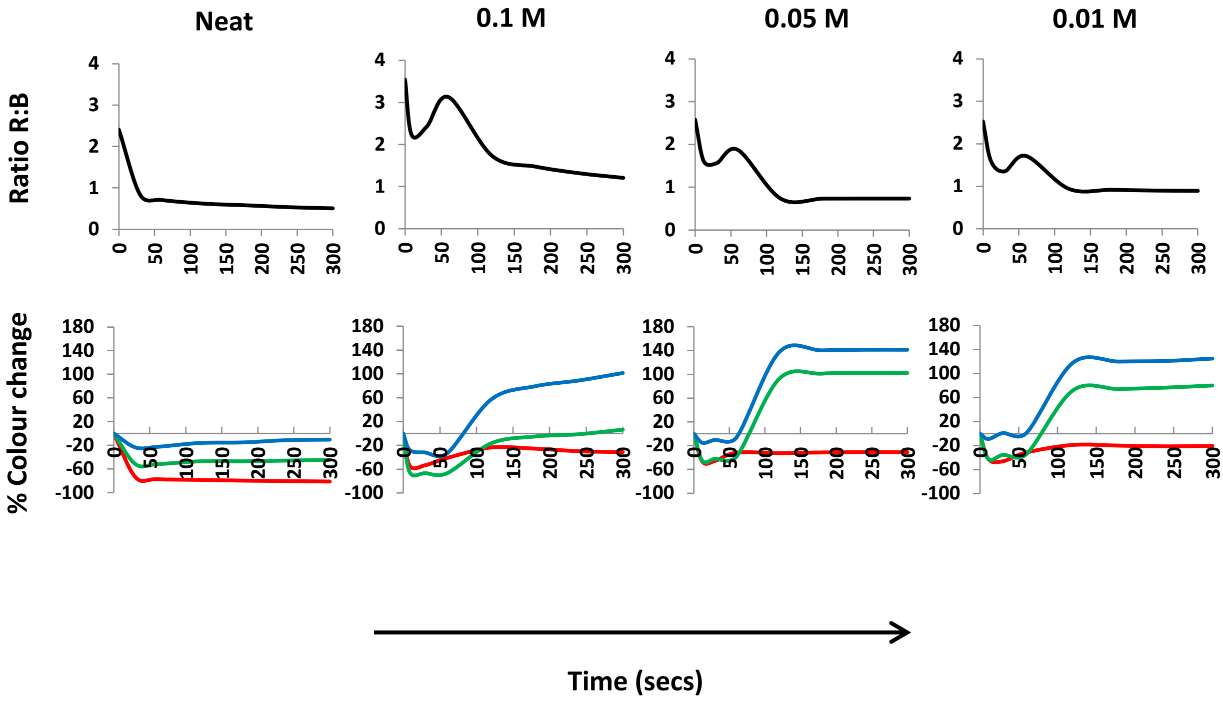

Figure S12: Colorimetric time response profiles over 5 minutes (300 seconds) by percentage change in each of the R, G and B colour components for TEP at varied concentrations.

TEphosphite, 254 nm

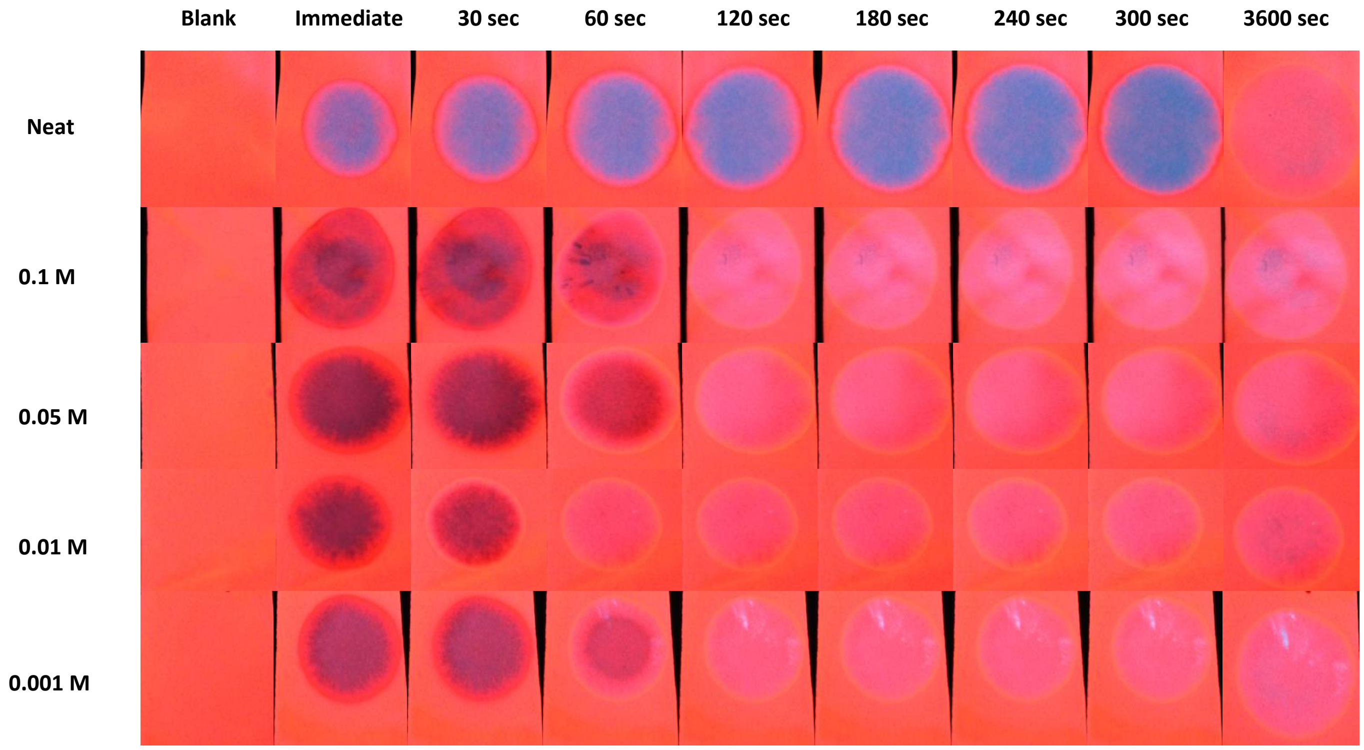

Figure S13: Normalised photographs of the dyad loaded paper assay with one drop of TEPhosphite at varied concentrations over time.

Table S6: RGB triplet data generated from the Spotfinder software for the normalised photographs from the TEPhosphite experiments in Figure S13.

		**0**	**11**	**30**	**60**	**120**	**180**	**240**	**300**	**3600**
**Neat**	**Red**	4080	2416	2288	2240	2112	2000	1920	1728	4032
	**Green**	1440	1616	1760	1856	1872	1936	1920	1792	1408
	**Blue**	1152	2704	2864	2992	3024	3072	3056	2880	1904
**0.1 M**		**0**	**9**	**30**	**60**	**120**	**180**	**240**	**300**	**3600**
	**Red**	4080	2704	2624	3232	4080	4080	4080	4080	3888
	**Green**	1552	720	752	816	1616	696	1760	1776	1680
	**Blue**	1424	1280	1376	1392	2432	2512	2576	2608	2528
**0.05 M**		**0**	**11**	**30**	**60**	**120**	**180**	**240**	**300**	**3600**
	**Red**	4080	2400	2592	3424	4080	4080	4080	4080	4080
	**Green**	1536	592	624	688	1408	1360	1376	1408	1296
	**Blue**	1280	1120	1168	1120	2048	1968	1984	2032	1904
**0.01 M**		**0**	**20**	**30**	**60**	**120**	**180**	**240**	**300**	**3600**
	**Red**	4080	2496	3088	4080	4080	4080	4080	4080	3984
	**Green**	1140	544	624	1136	1184	1200	1328	1344	1168
	**Blue**	1264	1024	1104	1648	1712	1712	1952	1952	1744
**0.001 M**		**0**	**18**	**30**	**60**	**120**	**180**	**240**	**300**	**3600**
	**Red**	4080	2944	3056	3696	4080	4080	4080	4080	4080
	**Green**	1344	880	912	976	1488	1520	1520	1552	1520
	**Blue**	1200	1504	1552	1520	2064	2081	2096	2112	2224

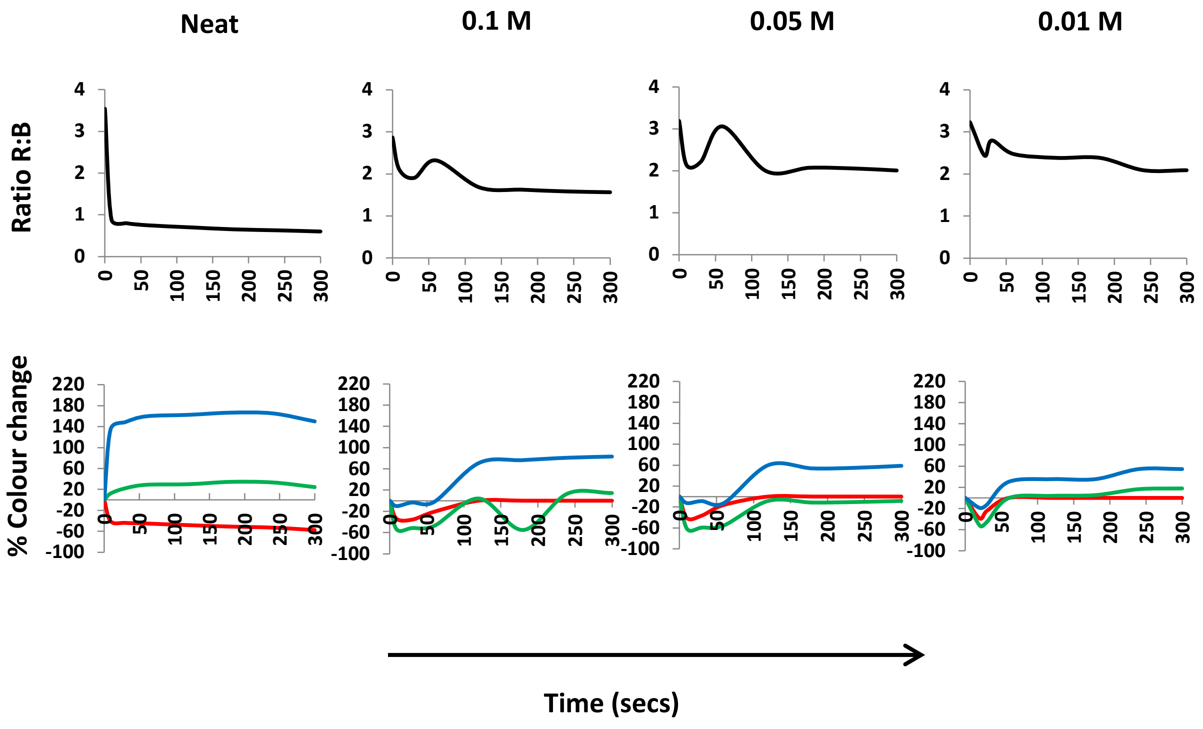

Figure S14: Colorimetric time response profiles over 5 minutes (300 seconds) by percentage change in each of the R, G and B colour components for TEPhosphite at varied concentrations.

TEA, 254 nm

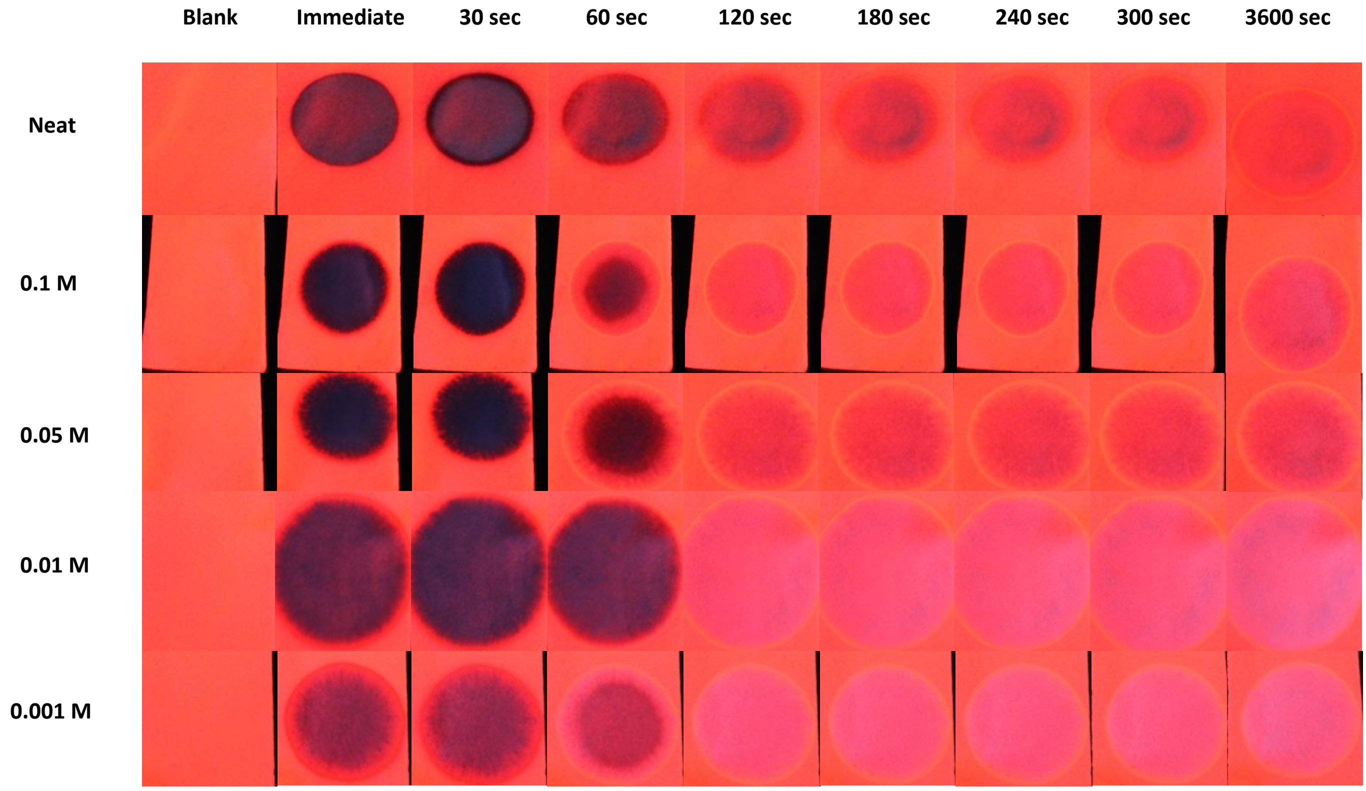

Figure S15: Normalised photographs of the dyad loaded paper assay with one drop of TEA at varied concentrations over time.

Table S7: RGB triplet data generated from the Spotfinder software for the normalised photographs from the TEA experiments in Figure S15.

**Neat**		**0**	**9**	**30**	**60**	**120**	**180**	**240**	**300**	**3600**
	**Red**	4080	3032	1744	2288	3184	3536	3728	3776	4000
	**Green**	1456	592	688	576	704	752	816	832	816
	**Blue**	1072	912	1056	880	992	1024	1072	1072	944
**0.1 M**		**0**	**9**	**30**	**60**	**120**	**180**	**240**	**300**	**3600**
	**Red**	4080	800	528	2128	4080	4080	4080	4080	3984
	**Green**	1520	432	416	368	944	992	992	1024	1008
	**Blue**	1200	880	912	672	1264	1312	1344	1392	1488
**0.05 M**		**0**	**10**	**30**	**60**	**120**	**180**	**240**	**300**	**3600**
	**Red**	4080	576	512	1904	4016	4035	4048	4048	3968
	**Green**	1552	384	368	240	880	880	912	912	912
	**Blue**	1216	816	752	432	1056	1088	1104	1152	1248
**0.01 M**		**0**	**20**	**30**	**60**	**120**	**180**	**240**	**300**	**3600**
	**Red**	4080	1776	1552	1888	4080	4080	4080	4080	4080
	**Green**	1424	544	576	624	1264	1296	1344	1296	1456
	**Blue**	1248	1040	1136	1200	1792	1840	1920	1888	2144
**0.001 M**		**0**	**18**	**30**	**60**	**120**	**180**	**240**	**300**	**3600**
	**Red**	4080	2352	2448	3200	4080	4080	4080	4080	4000
	**Green**	1392	592	640	720	1296	1344	1360	1392	1440
	**Blue**	1200	1088	1200	1232	1952	1984	2000	2048	2160

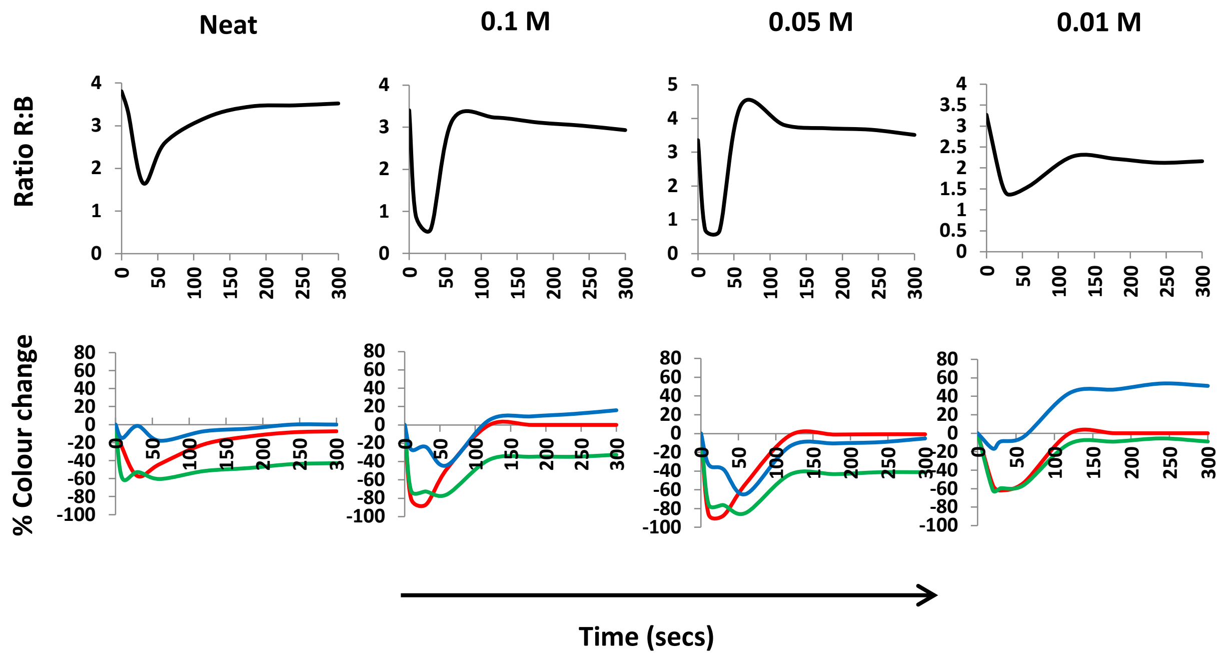

Figure S16: Colorimetric time response profiles over 5 minutes (300 seconds) by percentage change in each of the R, G and B colour components for TEA at varied concentrations.

INTERFERENTS, 254 nm

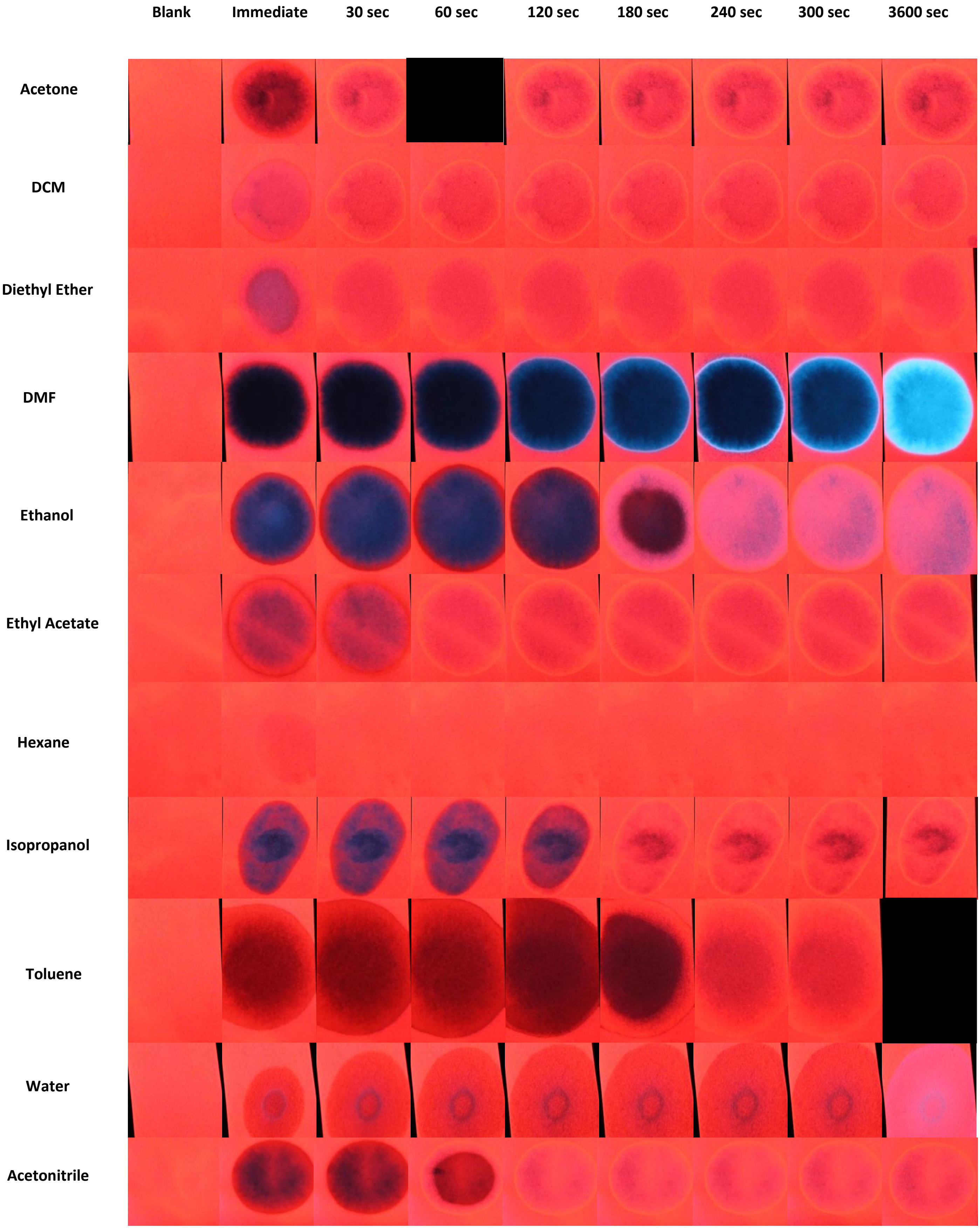

Figure S17: Normalised photographs of the dyad loaded paper assay with one drop of neat various solvent / interferent over time. The images of acetone at 1 minute (60 seconds) and toluene at 5 minutes (300 seconds) failed or were corrupted and have not been displayed.

Table S8: RGB triplet data generated from the Spotfinder software for the normalised photographs from the interferent experiments in Figure S17.

**Acetone**	**0**	**5**	**30**	**60**	**120**	**180**	**240**	**300**	**3600**	**0**
	4080	2432	3968	[redacted]	3968	3920	3952	3968	3872	4080
	1376	304	864	[redacted]	864	816	848	880	768	1376
	1216	576	1232	[redacted]	1216	1184	1200	1232	1104	1216
**DCM**		**0**	**9**	**30**	**60**	**120**	**180**	**240**	**300**	**3600**
	**Red**	4080	3920	3968	3984	3984	4000	4000	3984	4016
	**Green**	1168	880	848	848	864	880	880	864	896
	**Blue**	1040	1360	1088	1104	1120	1136	1152	1120	1168
**Diethyl Ether**		**0**	**11**	**30**	**60**	**120**	**180**	**240**	**300**	**3600**
	**Red**	4080	3088	3936	3952	3936	3952	3952	3952	4000
	**Green**	1216	864	800	816	800	816	832	832	880
	**Blue**	1072	1520	1104	1104	1072	1104	1104	1104	1168
**DMF**		**0**	**20**	**30**	**60**	**120**	**180**	**240**	**300**	**3600**
	**Red**	4080	224	224	208	192	176	176	160	432
	**Green**	1296	192	192	240	368	512	352	704	2944
	**Blue**	1200	400	432	528	848	1152	832	1440	3920
**Ethanol**		**0**	**12**	**30**	**60**	**120**	**180**	**240**	**300**	**3600**
	**Red**	4080	736	688	704	896	1456	3824	3856	3728
	**Green**	1456	816	736	688	608	400	1376	1424	1440
	**Blue**	1248	1712	1584	1488	1312	784	2176	2224	2272
**Ethyl Acetate**		**0**	**10**	**30**	**60**	**120**	**180**	**240**	**300**	**3600**
	**Red**	4080	2752	2928	3984	3904	3952	3968	3968	4032
	**Green**	1328	592	640	848	768	832	848	864	944
	**Blue**	1152	1104	1136	1168	1024	1104	1120	1136	1200
**Hexane**		**0**	**10**	**30**	**60**	**120**	**180**	**240**	**300**	**3600**
	**Red**	4080	4080	4080	4080	4080	4080	4080	4080	4080
	**Green**	1072	1088	1200	1200	1168	1152	1152	1136	1104
	**Blue**	1024	1120	1120	1120	1088	1088	1088	1088	1040
**Isopropanol**		**0**	**10**	**30**	**60**	**120**	**180**	**240**	**300**	**3600**
	**Red**	4080	928	880	912	1344	3488	3520	3360	3728
	**Green**	1152	640	640	656	592	576	592	528	672
	**Blue**	1008	1360	1392	1392	1200	880	864	816	960
**Toluene**		**0**	**18**	**30**	**60**	**120**	**180**	**240**	**300**	**3600**
	**Red**	4080	2224	2032	2160	1616	1392	3504	3664	[redacted]
	**Green**	1264	160	144	176	176	224	528	576	[redacted]
	**Blue**	1152	272	256	304	336	400	752	800	[redacted]
**Water**		**0**	**18**	**30**	**60**	**120**	**180**	**240**	**300**	**3600**
	**Red**	4080	3584	3376	3392	3392	3472	3408	3504	4016
	**Green**	1200	640	608	592	592	624	608	640	1552
	**Blue**	1216	880	912	880	880	912	896	944	2304
**Acetonitrile**		**0**	**18**	**30**	**60**	**120**	**180**	**240**	**300**	**3600**
	**Red**	4080	2192	2448	3152	4080	4080	4080	4080	4080
	**Green**	1376	416	384	384	1168	1152	1136	1120	1088
	**Blue**	1200	752	672	560	1344	1328	1328	1296	1280

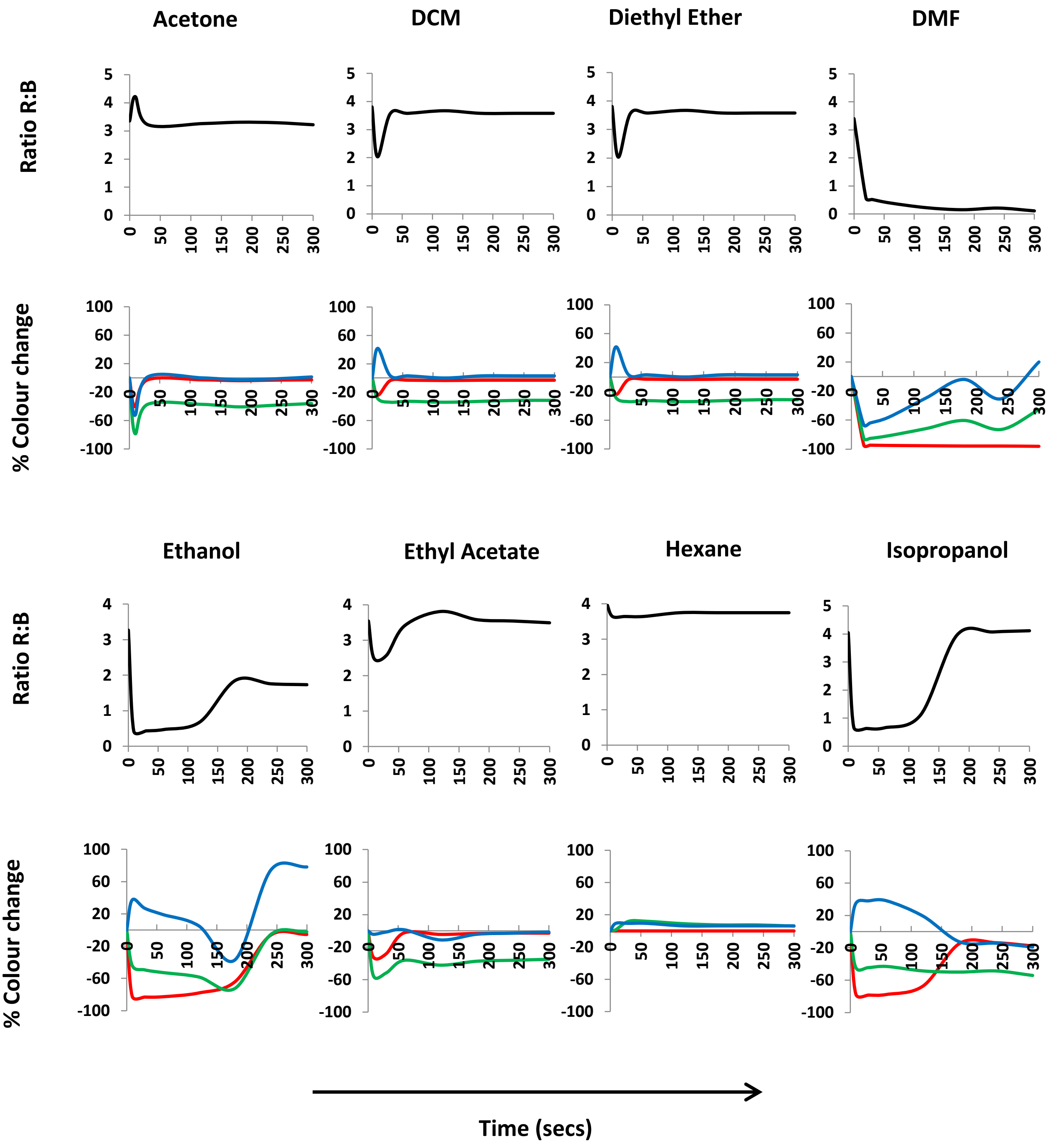

Figure S18a: Colorimetric time response profiles over 5 minutes (300 seconds) by percentage change in each of the R, G and B colour components for the interferents at varied concentrations.

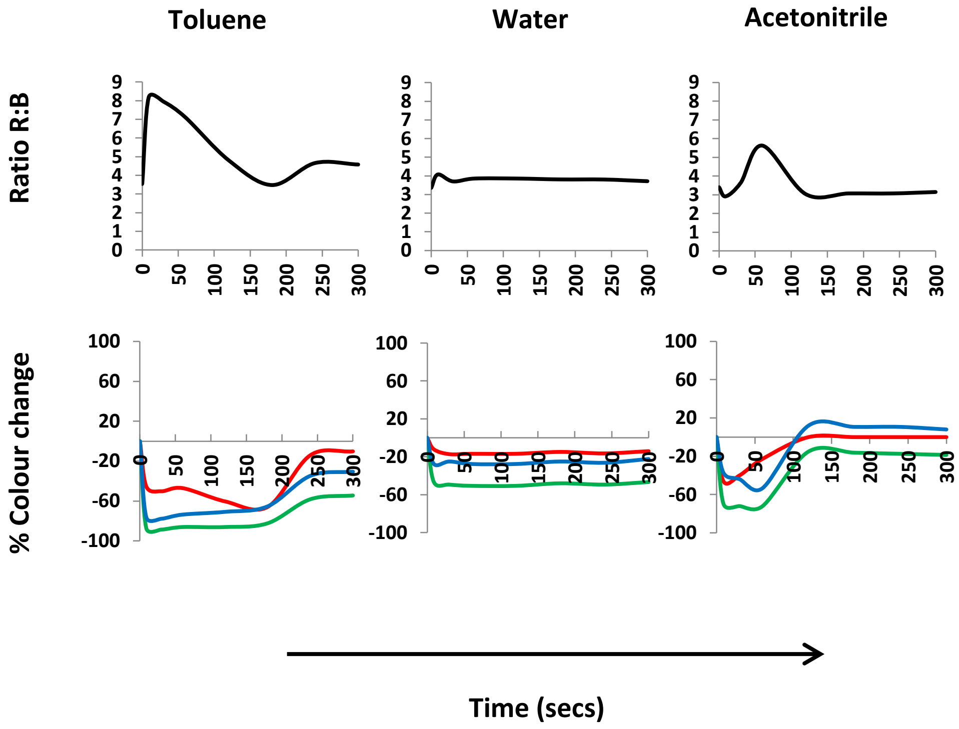

Figure S18b: Colorimetric time response profiles over 5 minutes (300 seconds) by percentage change in each of the R, G and B colour components for the interferents at varied concentrations.

References

1. G. H. Dennison, C. G. Bochet, C. Curty, J. Ducry, D. J. Nielsen, M.R. Sambrook, A. Zaugg and M. R. Johnston. *Eur. J. Inorg. Chem.*, 2016, **2016**, 1348-1358.
2. (a) D. Sykes, I. S. Tidmarsh, A. Barbieri, I. V. Sazanovich, J. A. Weinstein and M. D. Ward, *Inorg. Chem.*, 2011, **50**, 11323; (b) Q. Zhao, Y. Liu, Y. Cao, W. Lv, Q. Yu, S. Liu, X. Liu, M. Shi and W. Huang, *Adv. Opt. Mater.,* 2015, **3**, 233-240; (c) M. D. Ward, *Coord. Chem. Rev*., 2007, **251**, 1663-1677; (d) P. Coppo, M. Duati, V. N. Kozhevnikov, J. W. Hofstraat and L. De Cola, *Angew. Chem., Int. Ed.,* 2005, **44**, 1806-1810; (e) M. D. Ward, *Coord. Chem. Rev.,* 2010, **254**, 2634-2642.
3. (a) G. H. Dennison, M. R. Sambrook and M. R. Johnston. *Chem. Commun.*, 2014, **50**, 195-197; (b) G. H. Dennison, M. R. Sambrook and M. R. Johnston. RSC Adv., 2014, **4**, 55524-5552.
4. Schindelin, J.; Arganda-Carreras, I. & Frise, E. et al. "Fiji: an open-source platform for biological-image analysis", Nature Methods, 2012 **9**(7), 676-682.
5. SpotFinder (Specific Technologies, Mountain View, CA) C. L. Lonsdale, B.Taba, N. Queralto, R, A. Lukaszewski, R, A. Martino, P. A. Rhodes and S. H. Lim, PLOS ONE, *2013*, **8**, e62726. https://journals.plos.org/plosone/article?id=10.1371/journal.pone.0062726
